# No evidence for nudging after adjusting for publication bias

**DOI:** 10.1073/pnas.2200300119

**Published:** 2022-07-19

**Authors:** Maximilian Maier, František Bartoš, T. D. Stanley, David R. Shanks, Adam J. L. Harris, Eric-Jan Wagenmakers

**Affiliations:** ^a^Department of Experimental Psychology, University College London, London WC1H 0AP, United Kingdom;; ^b^Department of Psychological Methods, University of Amsterdam, Amsterdam, 1018 WS, The Netherlands;; ^c^Deakin Laboratory for the Meta-Analysis of Research, Deakin University, Burwood, VIC 3125, Australia;; ^d^Department of Economics, Deakin University, Burwood, VIC 3125, Australia

Thaler and Sunstein’s “nudge” (1) has spawned a revolution in behavioral science research. Despite its popularity, the “nudge approach” has been criticized for having a “limited evidence base” (e.g., ref. 2). Mertens et al. (3) seek to address that limitation with a timely and comprehensive metaanalysis. Mertens et al.’s headline finding is that “choice architecture [nudging] is an effective and widely applicable behavior change tool” (p. 8). We propose their finding of “moderate publication bias” (p. 1) is the real headline; when this publication bias is appropriately corrected for, no evidence for the effectiveness of nudges remains (Fig. 1).

**Fig. 1.**
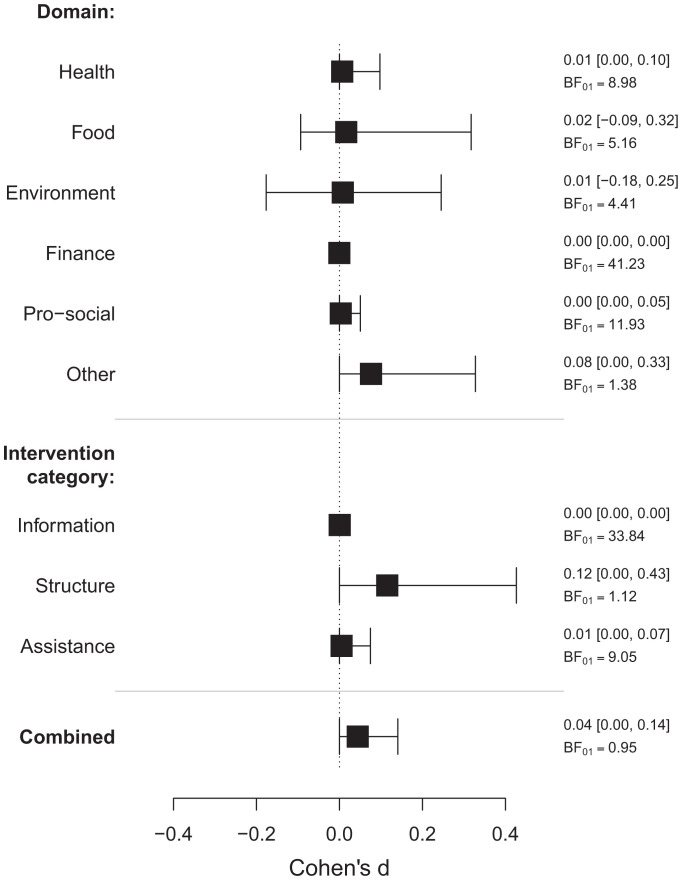
RoBMA_PSMA_ model-averaged posterior mean effect size estimates with 95% credible intervals and Bayes factors for the absence of the effect for the combined sample or split by either the domain or intervention category (ignoring the clustering of SEs). BF_01_ quantifies evidence for the null hypothesis. BF_01_ larger than one corresponds to evidence in favor of the null hypothesis, and BF_01_ lower than one corresponds to evidence in favor of the alternative hypothesis (evidence for the alternative hypothesis can be obtained by reciprocating the Bayes factor; BF_10_ = 1/BF_01_). As a rule of thumb, Bayes factors between 3 and 10 indicate moderate evidence, and Bayes factors larger than 10 indicate strong evidence.

Mertens et al. (3) find significant publication bias, through Egger regression. Their sensitivity analysis (4) indicates that the true effect size could be as low as *d* = 0.08 (if publication bias is severe). Mertens et al. argue that severe publication bias is only partially supported by the funnel plot and proceed largely without taking publication bias into account in their subsequent analyses. However, the reported Egger coefficient (*b* = 2.10) is “severe” (5).

A newly proposed bias correction technique, robust Bayesian metaanalysis (RoBMA) (6), avoids an all-or-none debate over whether or not publication bias is “severe.” RoBMA simultaneously applies 1) selection models that estimate relative publication probabilities (7) and 2) models of the relationship between effect sizes and SEs [i.e., Precision Effect Test and Precision Effect Estimate with Standard Error (6, 8, 9)]. Multimodel inference is then guided mostly by those models that predict the observed data best (6, 9, 10). RoBMA makes multimodel inferences about the presence or absence of an effect, heterogeneity, and publication bias (6, 9).

Table 1 compares the unadjusted results to the publication bias–adjusted results.* Since publication bias–corrected three-level selection models are computationally intractable, we analyzed the data in two ways: 1) ignoring the three-level structure (column 2) and 2) using only the most precise estimate from studies with multiple results (column 3). Strikingly, there is an absence of evidence for an overall effect and evidence against an effect in the “information” and “assistance” intervention categories, whereas the evidence is undecided for “structure” interventions. When using only the most precise estimates, we further find evidence against an effect in most of the domains, apart from “other,” “food,” and “prosocial” (the evidence is indecisive) and weak evidence for the overall effect.^†^ However, all intervention categories and domains apart from “finance” show evidence for heterogeneity, which implies that some nudges might be effective, even when there is evidence against the mean effect. Finally, we find strong evidence for publication bias across all subdomains (BF_pb_ > 10), apart from food, when using only the most precise estimates (BF_pb_ = 2.49).

**Table 1.** Comparison of unadjusted and adjusted effect size estimates for all studies and for subsets of studies based on different categories or domains

	Random effects	RoBMA_PSMA_	RoBMA_PSMA_ (precise)
Combined	0.43 [0.38, 0.48]	0.04 [0.00, 0.14]	0.11 [0.00, 0.24]
	*t*(333) = 16.51	BF_01_ = 0.95	BF_01_ = 0.31
Intervention category			
Information	0.25 [0.19, 0.30]	0.00 [0.00, 0.00]	0.00 [0.00, 0.07]
	*t*(88) = 8.79	BF_01_ = 33.84	BF_01_ = 10.57
Structure	0.58 [0.50, 0.66]	0.12 [0.00, 0.43]	0.23 [0.00, 0.49]
	*t*(186) = 13.93	BF_01_ = 1.12	BF_01_ = 0.33
Assistance	0.22 [0.15, 0.29]	0.01 [0.00, 0.07]	0.01 [0.00, 0.12]
	*t*(65) = 6.42	BF_01_ = 9.05	BF_01_ = 8.00
Domain			
Health	0.31 [0.22, 0.39]	0.01 [0.00, 0.10]	0.02 [0.00, 0.19]
	*t*(64) = 7.03	BF_01_ = 8.98	BF_01_ = 3.53
Food	0.66 [0.52, 0.81]	0.02 [−0.09, 0.32]	0.27 [0.00, 0.64]
	*t*(81) = 9.01	BF_01_ = 5.16	BF_01_ = 0.55
Environment	0.48 [0.37, 0.58]	0.01 [−0.18, 0.25]	0.00 [−0.44, 0.34]
	*t*(56) = 9.16	BF_01_ = 4.41	BF_01_ = 3.05
Finance	0.23 [0.15, 0.31]	0.00 [0.00, 0.00]	0.00 [0.00, 0.00]
	*t*(34) = 6.08	BF_01_ = 41.23	BF_01_ = 30.95
Prosocial	0.32 [0.22, 0.42]	0.00 [0.00, 0.05]	0.05 [0.00, 0.27]
	*t*(38) = 6.36	BF_01_ = 11.93	BF_01_ = 1.89
Other	0.40 [0.29, 0.50]	0.08 [0.00, 0.33]	0.04 [−0.22, 0.40]
	*t*(55) = 7.66	BF_01_ = 1.38	BF_01_ = 2.45

First column: Random effects metaanalysis estimates with 95% CI based on clustered SEs, all *P* values < 0.001. Second and third columns: RoBMA_PSMA_ model-averaged posterior mean effect size estimates with 95% credible intervals and Bayes factor for the presence of the effect ignoring the clustering of SEs or using the most precise estimates (precise). Results differ slightly from the moderator analysis presented in the article because we analyzed each subfield separately to allow 1) testing for the presence of the effect in each category/domain in the Bayesian framework, and 2) publication bias to operate differently in different subdomains.

We conclude that the “nudge” literature analyzed in ref. 3 is characterized by severe publication bias. Contrary to Mertens et al. (3), our Bayesian analysis indicates that, after correcting for this bias, no evidence remains that nudges are effective as tools for behaviour change.

## Data Availability

Data and analysis script are available in ref. 11.

## Footnotes

^*^Our analysis is based on the corrected dataset in ref. 12.

^†^We also reanalyzed the data by including only models of selection for statistical significance, confirming our results.
